# Rib Presence, Anterior Rib Cage Integrity, and Segmental Length Affect the Stability of the Human Thoracic Spine: An *in vitro* Study

**DOI:** 10.3389/fbioe.2020.00046

**Published:** 2020-02-07

**Authors:** Christian Liebsch, Hans-Joachim Wilke

**Affiliations:** Institute of Orthopaedic Research and Biomechanics, Trauma Research Centre Ulm, Ulm University, Ulm, Germany

**Keywords:** thoracic spine, rib cage, segmental flexibility, stepwise reduction, *in vitro* study, biomechanics, numerical model validation

## Abstract

The effects of segmental length as well as anterior rib cage and costovertebral joint integrity on thoracic spinal stability have not been extensively investigated, but are essential for the calibration and validation of numerical models of the thoracic spine and rib cage. The aim of the study was to quantify these effects by *in vitro* experiments. Eight human thoracic spine specimens (C7-L1) including the rib cage were loaded with pure moments of 5 Nm in flexion/extension, lateral bending, and axial rotation while tracking the motions of all functional spinal units. Specimens were tested stepwise in four different conditions: (1) In the intact condition, (2) after cutting all anterior rib-to-rib connections, (3) after partitioning the polysegmental specimens into monosegmental specimens, and (4) after removing the ribs in the monosegmental condition. Significant increases of the range of motion (*p* < 0.05) were especially found at the segmental levels of the upper half of the thoracic spine in all motion planes and for all resection steps, particularly in axial rotation, while the stabilizing effects of the structures decreased in inferior direction. Partitioning of polysegmental specimens into monosegmental specimens primarily affected the stability in lateral bending, while the effects of resection were generally lowest in flexion/extension. Presence of the ribs, anterior rib cage integrity, and segmental length all affect the thoracic spinal stability and have therefore to be considered in the calibration process of numerical models of the thoracic spine and rib cage.

## Introduction

The lumbar spine has been extensively studied in the past regarding the biomechanical effects of novel surgical techniques including the usage of rigid and flexible instrumentations. In case of the thoracic spine, however, these factors are not well-investigated and need to be considered separately due to the specific characteristics of the thoracic spinal morphology and the presence of the rib cage. Numerical models of the thoracic spine and rib cage can be used to simulate these effects in the treatment of specific thoracic spinal pathologies, such as scoliosis and hyperkyphosis. For the generation of reproducible and realistic results, numerical models of the thoracic spine and rib cage have to be calibrated and validated (Andriacchi et al., 1974; Sham et al., 2005; Schlager et al., 2018), preferably using experimental data from biomechanical *in vitro* tests.

The method of stepwise resection of anatomical structures was used in previous *in vitro* studies in order to create data for the calibration and validation of numerical models of the lumbar spine (Heuer et al., 2007). In case of the thoracic spine, motion data of monosegmental thoracic spinal specimens without ribs were generated for the validation of numerical models of the thoracolumbar spine (Wilke et al., 2017), while other studies used stepwise resection of rib cage structures to investigate the stabilizing effect of the thorax (Watkins et al., 2005; Brasiliense et al., 2011; Liebsch et al., 2017a). The quantitative effects of spinal length, intersegmental connection due to the anterior rib cage, and the stabilization by the ribs and costovertebral joints, however, have not yet been examined.

Therefore, the aim of this *in vitro* study was to determine possible effects of rib presence, sternal integrity, and specimen length by stepwise resection. It was hypothesized that all investigated structures contribute to thoracic spinal stability.

## Materials and Methods

### Specimens

Eight fresh frozen human thoracic spine specimens (C7-L1) including the intact rib cage were obtained from middle-aged male donors (Table 1). The specimens were inspected for signs of fractures, spinal deformities, tumors, and severe intervertebral disk degeneration prior to preparation by means of clinical CT scans (Siemens Somatom Definition AS, Siemens Healthcare, Erlangen, Germany) as well as for signs of ligamentous and cartilaginous injuries during preparation. All muscle and fat tissue was removed using surgical instruments, keeping all biomechanically relevant bony, cartilaginous, and ligamentous structures intact. For stability testing, the upper half of the cranial and the lower half of the caudal vertebrae were each embedded in polymethylmethacrylate (PMMA, Technovit 3040, Heraeus Kulzer, Wehrheim, Germany). During potting, care was taken to ensure full mobility of all ribs. To increase the stability of the embedding, screws were inserted in the respective vertebrae beforehand. Flanges were finally fixed concentrically to the cylindrical embeddings for load application. The specimens were stored at −20°C, prepared and tested at room temperature and periodically moistened with 0.9% saline solution while the overall preparation and testing period was kept below 20 h to avoid specimen decomposition. Prior to preparation and testing, the specimens were thawed for 12 h at 5°C.

**TABLE 1:** Data on donors specifying the thoracic spinal specimens which were used for the present *in vitro* study.

Donor no.	Age range	BMD	Body height	BMI
	(years)	(mgHA/cm^3^)	(cm)	(kg/m^2^)
1	36–40	129	183	23
2	50–55	172	185	18
3	50–55	63	183	14
4	50–55	126	175	19
5	56–60	91	188	21
6	56–60	123	168	24
7	56–60	36	178	25
8	66–70	91	183	21
Mean ± SD	56 ± 7	104 ± 40	180 ± 6	21 ± 3

*BMD, bone mineral density; BMI, body mass index; SD, standard deviation.*

### Experimental Design

**FIGURE 1:**
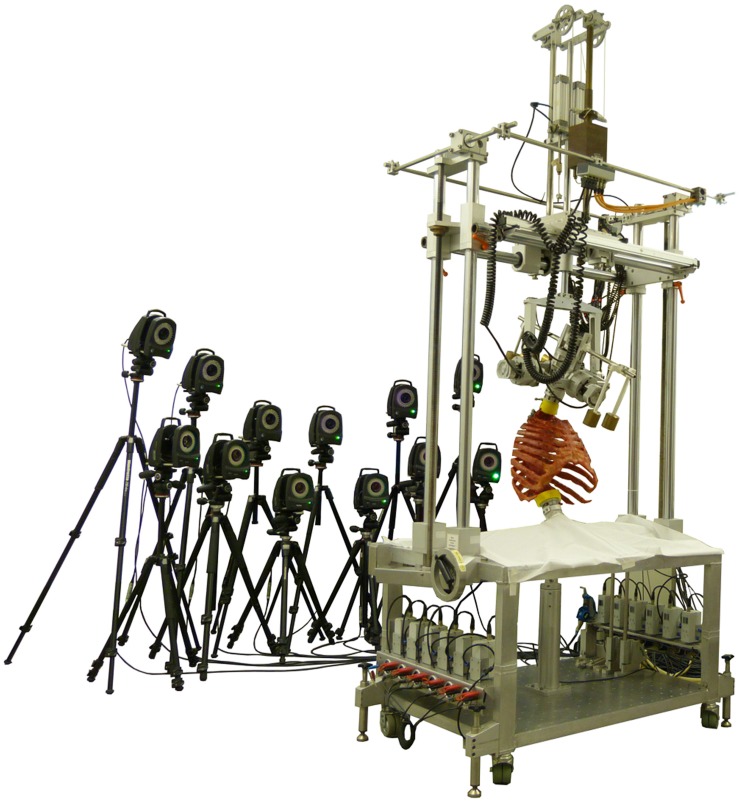
Illustration of the test setup showing a rib cage specimen within the spine tester surrounded by the optical motion tracking system consisting of 12 cameras.

Biomechanical testing was performed using a well-established universal spine tester (Figure 1) allowing almost unconstrained loading in all anatomical motion planes (Wilke et al., 1994). Specimens were loaded quasi-statically with pure moments of 5 Nm in the primary motion directions flexion, extension, left and right lateral bending, as well as left and right axial rotation. Loading was applied displacement-controlled to the upper end of the specimen with a constant rate of 1°/s for 3.5 loading cycles, of which the third full cycle was used for data evaluation to reduce viscoelastic effects (Wilke et al., 1998b), while the lower end was adjusted and rigidly fixed in the testing device. Simultaneously to spinal loading, segmental motions were measured using the optical motion tracking system Vicon MX13 (Vicon Motion Systems, Ltd., Oxford, United Kingdom) consisting of 12 cameras (Figure 1). For this purpose, three reflective markers were fixed to custom-made screws which were inserted in the bony portion of each spinous process to create local coordinate systems for each vertebra. Preliminary tests showed that the accuracy of this method has an average error less than 0.1°. Screw insertion was tried to be performed without affecting the natural flexibility of the single motion segments by leaving out all ligamentous structures.

**FIGURE 2:**
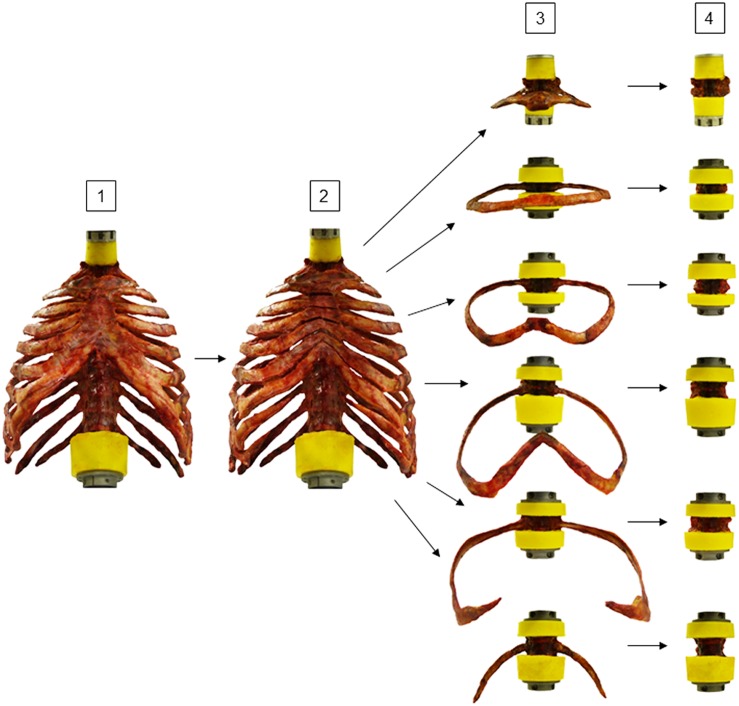
Illustration of experimental design. Eight rib cage specimens were tested stepwise [1] polysegmentally with intact sternum (Poly intact), [2] polysegmentally without intersegmental sternal and cartilaginous rib-to-rib connections (Poly w/o), [3] monosegmentally including ribs and sternal connection (Mono intact), and [4] monosegmentally without ribs (Mono w/o).

Thoracic spinal stability was tested stepwise in four different specimen conditions (Figure 2): in the first step, the polysegmental specimens (C7-L1) were loaded in the intact condition. In the second step, loading was performed after transversally cutting the sternal and all cartilaginous rib-to-rib connections at the intercostal levels using an oscillating bone saw (OR-SY-518.01, Synthes^®^, Zuchwil, Switzerland) and a scalpel to create anterior rib cage disconnections between the single spinal segments. In the third step, the polysegmental specimens were separated into the six thoracic spinal motion segments T1-T2, T3-T4, T5-T6, T7-T8, T9-T10, and T11-T12 by cutting the respective intervertebral disks and spinal ligaments using a scalpel. The monosegmental specimens were again embedded in PMMA and biomechanically tested afterward. In the fourth step, testing was performed after removing the ribs by carefully cutting the stabilizing ligaments at the costovertebral joint using a scalpel.

### Data Evaluation and Statistics

Data obtained from biomechanical loading and motion analysis were merged and evaluated regarding range of motion (ROM) and neutral zone (NZ) using a custom-written Matlab script (Matlab R2014b, MathWorks, Inc., Natick, MA, United States). Using Microsoft Excel (Microsoft, Corp., Redmond, WA, United States), data were compiled and prepared for statistical analysis, which was performed using the statistics software SPSS (SPSS 21, IBM, Corp., Armonk, NY, United States).

Due to the overall low, but for biomechanical studies usual sample size of *n* = 8, cumulated data are presented as median and range. Changes in the median ROM between single sequential testing steps were checked for significance using the non-parametric Friedman test for multiple paired samples, since non-normal distribution was expected based on the low sample size. The significance level was set to α = 0.05.

### Ethics Statement

The use of human thoracic spine specimens was approved by the Ethical Committee Board of the University of Ulm, Germany, on November 2014 (No. 302/14). The specimens were obtained from an accredited and ethically approved body donation program (Science Care, Inc., Phoenix, AZ, United States).

## Results

**FIGURE 3:**
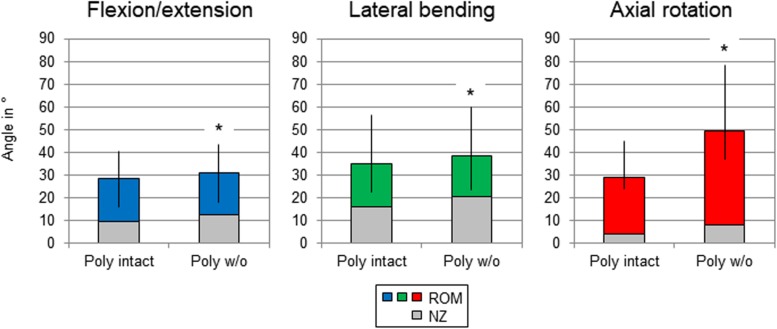
Graphical illustration of the results for total T1-T12 range of motion (ROM) and neutral zone (NZ) at pure moments of ± 5 Nm (*n* = 8) depending on primary motion plane and testing step. Significant differences compared to the previous condition (*p* < 0.05) are marked with an asterisk.

In general, the resections of all investigated structures affected the stability of the thoracic spine. After transversal cutting of the sternal and cartilaginous rib-to-rib connections, a significant increase (*p* < 0.05) of the ROM of the entire thoracic spine (T1-T12) was detected in all three motion planes (Figure 3 and Table 2). In flexion/extension, the lowest increase of the ROM with 9% on average (from 28 to 31°) as well as the overall lowest was ROM were detected both in the intact condition and after the first resection step. In lateral bending, where the ROM grew by 11% (from 35 to 39°), the highest ROM was found in the intact condition among all three motion planes. The largest ROM increase was detected in axial rotation by 72% on average from 29° in the intact condition to 50° after disconnecting the ribs.

**TABLE 2:** Results for total T1-T12 range of motion at pure moments of ± 5 Nm (*n* = 8) depending on primary motion plane and testing step.

Motion plane	Testing step [No.]	Median ROM in °	Maximum ROM in °	Minimum ROM in °	RC in %	*p*
Flexion/extension	Poly intact [1]	28.4	40.5	16.0		
	Poly w/o [2]	30.8	43.6	18.3	+9	**0.008**
Lateral bending	Poly intact [1]	35.0	56.4	22.6		
	Poly w/o [2]	38.8	59.9	23.6	+11	**0.008**
Axial rotation	Poly intact [1]	28.8	45.0	23.9		
	Poly w/o [2]	49.6	78.4	37.1	+72	**0.008**

**p-*Values < 0.05 are printed in bold. ROM, range of motion; RC, relative change of the median value compared to the previous condition; p, probability value.*

**FIGURE 4:**
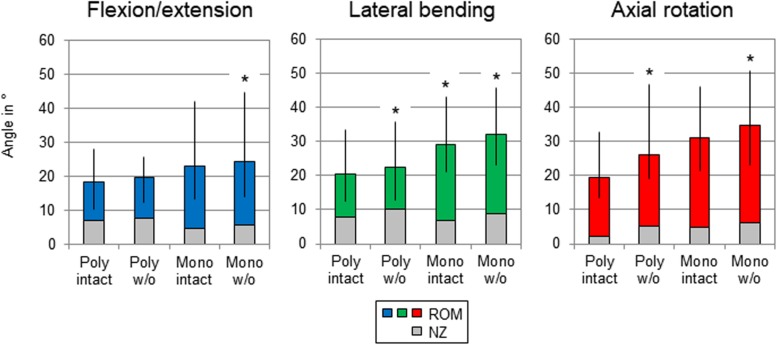
Graphical illustration of the results for T1-T12 ROM and NZ as sum of the six tested segmental levels (T1-T2 + T3-T4 + …) at pure moments of ± 5 Nm (*n* = 8) depending on primary motion plane and testing step. Significant differences compared to the previous condition (*p* < 0.05) are marked with an asterisk.

After summation of the ranges of motion of all tested spinal motion segments (i.e., T1-T2 + T3-T4 + …), the flexibility also increased after each resection step in all three motion planes (Figure 4 and Table 3). Transversal cutting of the sternal and cartilaginous rib-to-rib connections led to significant ROM increases in lateral bending (+10%) and axial rotation (+34%), while partitioning of polysegmental specimens into monosegmental specimens solely increased the ROM significantly in lateral bending (+30%). Significant increases were especially detected after resection of the ribs in the monosegmental state (flexion/extension: +6%, lateral bending: +9%, axial rotation: +12%).

**TABLE 3:** Results for T1-T12 range of motion as sum of the six tested segmental levels (T1-T2 + T3-T4 + …) at pure moments of ± 5 Nm (*n* = 8) depending on primary motion plane and testing step.

Motion plane	Testing step [No.]	Median ROM in °	Maximum ROM in °	Minimum ROM in °	RC in %	*p*
Flexion/extension	Poly intact [1]	18.5	27.9	10.5		
	Poly w/o [2]	19.8	25.9	12.5	+7	1.000
	Mono intact [3]	23.1	42.0	13.4	+17	0.070
	Mono w/o [4]	24.4	44.6	14.0	+6	**0.008**
Lateral bending	Poly intact [1]	20.5	33.5	12.4		
	Poly w/o [2]	22.4	35.7	12.8	+10	**0.008**
	Mono intact [3]	29.3	43.3	21.1	+30	**0.008**
	Mono w/o [4]	32.0	45.7	23.1	+9	**0.008**
Axial rotation	Poly intact [1]	19.5	32.7	13.4		
	Poly w/o [2]	26.1	46.7	19.1	+34	**0.008**
	Mono intact [3]	31.2	46.1	21.5	+20	0.070
	Mono w/o [4]	34.9	50.7	23.1	+12	**0.008**

**p-*Values < 0.05 are printed in bold. ROM, range of motion; RC, relative change of the median value compared to the previous condition; p, probability value.*

**FIGURE 5:**
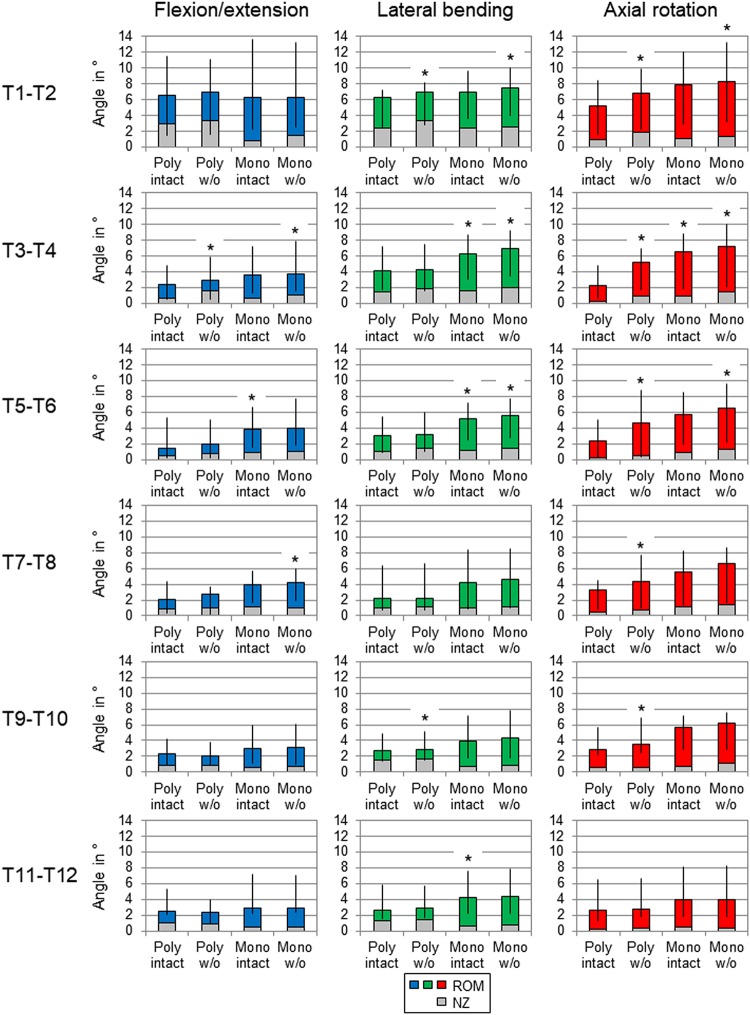
Graphical illustration of the results for ROM and NZ of the six tested segmental levels (T1-T2, T3-T4, …) at pure moments of ± 5 Nm (*n* = 8) depending on primary motion plane and testing step. Significant differences compared to the previous condition (*p* < 0.05) are marked with an asterisk.

Regarding the isolatedly viewed flexibility of the single thoracic functional spinal units, significant effects of the resection steps on the ranges of motion were especially found in the upper thoracic spinal section between T1-T2 and T5-T6 in all three motion planes (Figure 5 and Tables 4–6). In particular, these effects were seen after rib removal in the monosegmental state during lateral bending and axial rotation with average ROM increases of about 10%, respectively. Significant effects were further detected at these segmental levels after transversal cutting of the sternal and cartilaginous rib-to-rib connections in the polysegmental state during axial rotation, which were also found in the motion segments T7-T8 and T9-T10 (Figure 5 and Tables 7, 8), while the ROM was increased by about 60% on average in these motion segments. In flexion/extension, generally low effects of the single resection steps were identified, especially in the motion segments T1-T2 (Figure 5 and Table 4), T9-T10 (Figure 5 and Table 8), and T11-T12 (Figure 5 and Table 9), where no significant ROM increases were found. In lateral bending, significant effects of partitioning the polysegmental specimens into monosegmental specimens were detected in the motion segments T3-T4 (Figure 5 and Table 5), T5-T6 (Figure 5 and Table 6), and T11-T12 (Figure 5 and Table 9) with the ROM increasing by about 50% on average.

**TABLE 4:** Results for T1-T2 range of motion at pure moments of ± 5 Nm (*n* = 8) depending on primary motion plane and testing step.

Motion plane	Testing step [No.]	Median ROM in °	Maximum ROM in °	Minimum ROM in °	RC in %	*p*
Flexion/extension	Poly intact [1]	6.5	11.4	1.4		
	Poly w/o [2]	6.9	11.0	1.6	+6	0.289
	Mono intact [3]	6.3	13.7	2.2	−9	1.000
	Mono w/o [4]	6.2	13.3	2.5	±0	0.289
Lateral bending	Poly intact [1]	6.3	7.2	2.5		
	Poly w/o [2]	6.9	8.1	2.7	+9	**0.008**
	Mono intact [3]	6.9	9.5	3.6	±0	0.727
	Mono w/o [4]	7.4	10.0	4.0	+8	**0.008**
Axial rotation	Poly intact [1]	5.2	8.4	1.6		
	Poly w/o [2]	6.8	9.9	2.2	+31	**0.008**
	Mono intact [3]	7.9	12.1	2.9	+16	0.070
	Mono w/o [4]	8.3	13.3	3.2	+5	**0.008**

**p-*Values < 0.05 are printed in bold. ROM, range of motion; RC, relative change of the median value compared to the previous condition; p, probability value.*

**TABLE 5:** Results for T3-T4 range of motion at pure moments of ± 5 Nm (*n* = 8) depending on primary motion plane and testing step.

Motion plane	Testing step [No.]	Median ROM in °	Maximum ROM in °	Minimum ROM in °	RC in %	*p*
Flexion/extension	Poly intact [1]	2.4	4.7	0.4		
	Poly w/o [2]	3.0	5.8	0.5	+24	**0.008**
	Mono intact [3]	3.5	7.1	1.3	+19	0.070
	Mono w/o [4]	3.8	7.8	1.5	+7	**0.008**
Lateral bending	Poly intact [1]	4.1	7.2	1.6		
	Poly w/o [2]	4.2	7.5	1.6	+3	0.727
	Mono intact [3]	6.2	8.7	3.1	+47	**0.008**
	Mono w/o [4]	6.9	9.2	3.4	+12	**0.008**
Axial rotation	Poly intact [1]	2.2	4.8	0.6		
	Poly w/o [2]	5.2	6.9	1.7	+135	**0.008**
	Mono intact [3]	6.5	8.7	1.9	+25	**0.008**
	Mono w/o [4]	7.2	10.0	2.0	+11	**0.008**

**p-*Values < 0.05 are printed in bold. ROM, range of motion; RC, relative change of the median value compared to the previous condition; p, probability value.*

**TABLE 6:** Results for T5-T6 range of motion at pure moments of ± 5 Nm (*n* = 8) depending on primary motion plane and testing step.

Motion plane	Testing step [No.]	Median ROM in °	Maximum ROM in °	Minimum ROM in °	RC in %	*p*
Flexion/extension	Poly intact [1]	1.4	5.3	0.3		
	Poly w/o [2]	1.9	5.1	0.2	+35	1.000
	Mono intact [3]	3.9	6.7	1.5	+102	**0.008**
	Mono w/o [4]	4.0	7.7	1.8	+3	0.727
Lateral bending	Poly intact [1]	3.1	5.4	0.9		
	Poly w/o [2]	3.1	5.9	1.0	+1	0.070
	Mono intact [3]	5.1	7.2	2.5	+65	**0.008**
	Mono w/o [4]	5.6	7.8	2.7	+9	**0.008**
Axial rotation	Poly intact [1]	2.3	5.0	0.2		
	Poly w/o [2]	4.6	8.7	0.3	+97	**0.008**
	Mono intact [3]	5.7	8.5	1.9	+23	0.070
	Mono w/o [4]	6.6	9.5	2.3	+15	**0.008**

**p-*Values < 0.05 are printed in bold. ROM, range of motion; RC, relative change of the median value compared to the previous condition; p, probability value.*

**TABLE 7:** Results for T7-T8 range of motion at pure moments of ± 5 Nm (*n* = 8) depending on primary motion plane and testing step.

Motion plane	Testing step [No.]	Median ROM in °	Maximum ROM in °	Minimum ROM in °	RC in %	*p*
Flexion/extension	Poly intact [1]	2.1	4.4	0.3		
	Poly w/o [2]	2.7	3.7	0.3	+31	1.000
	Mono intact [3]	3.9	5.7	1.7	+45	0.070
	Mono w/o [4]	4.2	6.0	1.9	+7	**0.008**
Lateral bending	Poly intact [1]	2.2	6.3	0.7		
	Poly w/o [2]	2.2	6.7	0.7	−2	0.289
	Mono intact [3]	4.2	8.3	0.9	+93	0.070
	Mono w/o [4]	4.7	8.5	1.0	+10	0.289
Axial rotation	Poly intact [1]	3.2	4.5	0.8		
	Poly w/o [2]	4.3	7.7	1.0	+33	**0.008**
	Mono intact [3]	5.6	8.2	1.4	+30	0.070
	Mono w/o [4]	6.6	8.6	1.6	+19	0.070

**p-*Values < 0.05 are printed in bold. ROM, range of motion; RC, relative change of the median value compared to the previous condition; p, probability value.*

**TABLE 8:** Results for T9-T10 range of motion at pure moments of ± 5 Nm (*n* = 8) depending on primary motion plane and testing step.

Motion plane	Testing step [No.]	Median ROM in °	Maximum ROM in °	Minimum ROM in °	RC in %	*p*
Flexion/extension	Poly intact [1]	2.3	4.1	1.0		
	Poly w/o [2]	2.0	3.8	1.0	−14	0.727
	Mono intact [3]	3.0	6.0	1.1	+48	0.070
	Mono w/o [4]	3.1	6.0	0.9	+4	0.289
Lateral bending	Poly intact [1]	2.7	4.8	1.4		
	Poly w/o [2]	2.9	5.1	1.5	+8	**0.008**
	Mono intact [3]	3.9	7.2	1.8	+36	0.070
	Mono w/o [4]	4.3	7.8	1.8	+9	0.070
Axial rotation	Poly intact [1]	2.9	5.7	2.2		
	Poly w/o [2]	3.5	6.9	2.5	+22	**0.008**
	Mono intact [3]	5.7	7.2	2.8	+62	0.070
	Mono w/o [4]	6.2	7.5	2.9	+9	0.070

**p-*Values < 0.05 are printed in bold. ROM, range of motion; RC, relative change of the median value compared to the previous condition; p, probability value.*

**TABLE 9:** Results for T11-T12 range of motion at pure moments of ± 5 Nm (*n* = 8) depending on primary motion plane and testing step.

Motion plane	Testing step [No.]	Median ROM in °	Maximum ROM in °	Minimum ROM in °	RC in %	*p*
Flexion/extension	Poly intact [1]	2.4	5.3	2.1		
	Poly w/o [2]	2.4	4.0	1.0	−4	1.000
	Mono intact [3]	2.8	7.1	2.2	+20	0.727
	Mono w/o [4]	2.8	7.0	2.4	±0	0.289
Lateral bending	Poly intact [1]	2.6	5.8	1.5		
	Poly w/o [2]	2.9	5.7	1.7	+13	0.289
	Mono intact [3]	4.2	7.5	2.2	+43	**0.008**
	Mono w/o [4]	4.3	7.8	2.3	+4	0.070
Axial rotation	Poly intact [1]	2.7	6.5	1.3		
	Poly w/o [2]	2.7	6.6	1.8	+4	0.289
	Mono intact [3]	4.0	8.1	1.8	+44	0.289
	Mono w/o [4]	4.0	8.3	1.8	+1	0.125

**p-*Values < 0.05 are printed in bold. ROM, range of motion; RC, relative change of the median value compared to the previous condition; p, probability value.*

Neutral zone values of the single thoracic spinal motion segments generally exhibited too high variability for conclusive statistical analysis, particularly in the polysegmental setup. Overall, NZ values were highest in the polysegmental state after transversal cutting of the sternal and cartilaginous rib-to-rib connections in flexion/extension and lateral bending, respectively, while increasing almost constantly after every resection step in axial rotation. The results for all ROM and NZ values, together with statistical analysis values, are retrievable from the Supplementary Material file attached to the electronic version of this publication.

## Discussion

The stabilizing effect of the single rib cage structures is still not fully understood. For the highest possible accuracy of numerical model calibration and validation, but also regarding the interpretation of *in vitro* studies on the thoracic spine and the clinical application of novel treatment methods, data on these stabilizing effects are essential. Therefore, this study aimed to quantify possible effects of anterior rib cage disconnection, reduction of spinal length, and removal of the ribs on the segmental stability of the thoracic spine.

The results of the present study indicate that the upper rib cage section primarily affects thoracic spinal stability, while the stabilizing effect of the rib cage generally decreases in inferior direction. This can be explained by the more rigid anterior connection of the ribs in the upper half of the bony thorax due to the sternal complex, while becoming more flexible in the lower half because of solely cartilaginous connections in case of the false ribs as well as missing connections in case of the floating ribs. This effect was especially seen after removal of the ribs, showing significant ROM increase in the motion segments T1-T2 to T5-T6, but not in the motion segments T7-T8 to T11-T12. Therefore, it can be assumed that the integrity of the sternal complex represents a major factor in the stability of the thoracic spine, which corresponds to the results of previous *in vitro* studies investigating the effects of median sternotomy (Brasiliense et al., 2011; Liebsch et al., 2017b), transversal sternotomy (Horton et al., 2005), and transversal sternal fracture (Watkins et al., 2005) on thoracic spinal stability. As a consequence, surgical releases of sternal structures should be rigidly fixed intraoperatively to avoid structural overstress and painful pseudarthrosis in the anterior rib cage as well as post-operative destabilization of the spine.

The highest impact of rib cage resection on thoracic spinal stability was generally found in axial rotation movement, especially in the upper rib cage half and after transversally cutting the sternal and cartilaginous rib-to-rib connections. This indicates that the rib cage mainly stabilizes the thoracic spine in the transversal plane, which was also detected in previous *in vitro* studies investigating the mechanical contribution of the rib cage on thoracic spinal stability (Mannen et al., 2015, 2018; Liebsch et al., 2017a; Anderson et al., 2018). This effect can be explained by the specific morphology of the rib cage, generally allowing forward and backward bending as well as sideward bending rather than axial rotation movement due to an increased torsional resistance in this motion plane.

Significant effects of segmental length were mainly found in lateral bending movement, indicating that especially sideward bending resistance depends on spinal structures which extend across multiple segments. From a biomechanical point of view, laterally positioned ligaments, such as the intertransverse or costotransverse ligaments, could account for higher spinal stability due to the increased lever arm in the transversal plane, while their stabilizing effect is probably enhanced by increasing spinal length and intact costovertebral joints. In general, the effect of segmental length on thoracic spinal stability corresponds with findings of a previous *in vitro* study investigating the influence of specimen length on the ROM in the lumbar spine (Kettler et al., 2000). The destabilizing effects of rib head release, which is used in the surgical treatment of scoliosis, has already been shown in previous *in vitro* studies using polysegmental (Feiertag et al., 1995; Liebsch et al., 2017a) as well as monosegmental (Oda et al., 2002) experimental designs. The present study, however, was the first to show that rib head release primarily affects the upper rib cage half and mainly lateral bending as well as axial rotation movements, while the effect was generally decreasing in inferior direction, which could also be attributed to the more rigid anterior connection of the upper ribs. Therefore, it can be assumed that rib head release in the upper and middle section of the thoracic spine has a higher destabilizing effect than in the lower thoracic spine, potentially having higher impact in the surgical correction of spinal deformities. In contrast, rib head release in the lower thoracic spine could thus be contraindicated.

Comparing the results of the present study with data from previous studies, the segmental ranges of motion were overall similar to the results of *in vivo* as well as *in vitro* studies regarding qualitative intersegmental motion distribution along the thoracic spine. Quantitatively, the ranges of motion were slightly lower using the polysegmental setup and slightly higher using the monosegmental setup compared to average ROM data summarized in a literature review on thoracic spinal motion segments including multiple different *in vitro* test setups and tested segmental levels (Borkowski et al., 2016), while being distinctly lower compared to a further *in vitro* study investigating the flexibility of all thoracic spinal motion segments for the validation of thoracolumbar numerical models (Wilke et al., 2017). These quantitative differences most probably can be attributed to the applied loads, since Wilke and colleagues, used pure moments of 7.5 Nm in order to facilitate comparisons with *in vitro* studies on the lumbar spine, while the analyzed studies in the literature review of Borkowski et al. (2016), contained loading setups using pure moments between 1.5 and 8 Nm, whereas pure moments of 5 Nm were used in the present study, generally making conclusive quantitative comparisons difficult. Compared to the results of *in vivo* studies in flexion/extension (Morita et al., 2014), lateral bending (Fujimori et al., 2014), and axial rotation (Fujimori et al., 2012), the results of the present study showed overall equivalent segmental ROM in case of the polysegmental setup with intact rib cage, indicating that this setup might be most suitable for simulating quasi-physiological loading *in vitro*. Pure moment loading therefore seems to represent a limited, however adequate approach for numerical model calibration and validation of the thoracic spine and rib cage. Although the simulation of muscle forces and body weight would probably represent more physiological loading conditions, thoracic spinal stiffness is significantly decreased *in vitro* and further boundary conditions are created for the numerical models (Liebsch et al., 2018). Moreover, pure moment application was shown to generate forces and moments in the lumbar spine that are comparable to the *in vivo* situation (Wilke et al., 2001). Nevertheless, future *in vitro* and *in silico* studies should also include the effect of compressive loading in addition to sole pure moment loading in order to generate realistic biomechanical behavior.

Due to the specific nature of experimental testing of biological materials, the present study entails several limitations. While it was shown that the chosen *in vitro* testing conditions did not significantly affect the flexibility of spinal specimens (Wilke et al., 1998a), the ROM values of the present study were generally subjected to high variations, most probably caused by the characteristic properties of the individual specimens. It was shown in previous studies that especially thoracic spinal motion segments are influenced by intervertebral disk degeneration including disk narrowing and osteophyte formation, potentially leading to reduced segmental ROM depending on age (Healy et al., 2015), which varied from 40 to 68 years for the donors in the present study (Table 1), as well as to altered thoracic spinal kinematics (Liebsch et al., 2019b). However, the primary intention of the present study was to generate ROM data of average thoracic spine segments within a reasonable margin regarding age. Moreover, specimens were checked for severely degenerated intervertebral disks before testing. Other specific parameters, such as sex, bone mineral density, body mass index, and body height are supposed not to directly affect the spinal ROM. Furthermore, the relative differences in ROM between the single resection steps should not be affected by these factors. Nevertheless, when calibrating or validating a numerical model using the data of the present study, these parameters should be taken into account regarding the inherent properties of the respective model, representing a male, mid-age thoracic spine including average bone mineral density, body height, and body mass index.

In the present study, a sample size of *n* = 8 was used, which is generally sufficient for the calibration and validation of numerical models and the interpretation of *in vitro* data. Compared to the sample size of clinical studies and considering certain data variability in the testing of biological materials, however, the sample size of the present *in vitro* study is too low to allow direct clinical conclusions. Therefore, the presented data including the significant differences between the single resection steps should primarily be used for the calibration and validation process of numerical models of the thoracic spine, which then can be used for the evaluation of novel surgical treatments. Statistical comparisons and interpretations of differences between the intact condition and both monosegmental conditions were not performed in this study due to the fact that resections were performed in different anatomical planes, making definite conclusions difficult. Moreover, multiple testing would have potentially caused cumulated α errors, which could have led to reduced statistical power after *post hoc* correction, especially regarding the small differences in ROM on segmental level.

Using the data presented in this study, numerical models can be calibrated more accurately by adapting their material properties to reproduce the experimentally determined ROM data. The data can be used either for polysegmental models with rib cage or monosegmental models with or without ribs in addition to data from previous *in vitro* studies using stepwise resection of single rib cage structures (Liebsch et al., 2017a) and functional structures of single thoracic spinal motion segments (Wilke et al., 2019), as well as data regarding relative motions between rib cage structures and the thoracic spine (Liebsch et al., 2019a). In case of polysegmental models, it is recommended to calibrate the model in reverse order of the resection steps, starting from monosegmental spinal units. The intact state could then be used for validation in order to achieve the highest model accuracy, since the present study showed that every resection step affected thoracic spinal stability.

## Data Availability Statement

The raw data supporting the conclusions of this article will be made available by the authors, without undue reservation, to any qualified researcher.

## Ethics Statement

The studies involving human participants were reviewed and approved by the Ethics Committee Board University of Ulm, Ulm, Germany; ethik-kommission@uni-ulm.de. Written informed consent for participation was not required for this study in accordance with the national legislation and the institutional requirements.

## Author Contributions

CL: study design, experimental testing, data analysis, and manuscript preparation. H-JW: funding acquisition, planning, discussion, and manuscript review.

## Conflict of Interest

The authors declare that the research was conducted in the absence of any commercial or financial relationships that could be construed as a potential conflict of interest.
